# Varying but reduced use of postoperative mobilization restrictions after primary total hip arthroplasty in Nordic countries: a questionnaire-based study

**DOI:** 10.1080/17453674.2019.1572291

**Published:** 2019-02-11

**Authors:** Kirill Gromov, Anders Troelsen, Maziar Modaddes, Ola Rolfson, Ove Furnes, Geir Hallan, Antti Eskelinen, Perttu Neuvonen, Henrik Husted

**Affiliations:** a Department of Orthopaedic Surgery, Copenhagen University Hospital Hvidovre, Denmark;; b Danish Hip Arthroplasty Registry;; c Department of Orthopaedics, Institute of Clinical Sciences, Sahlgrenska Academy, University of Gothenburg, Gothenburg, Sweden;; d Swedish Hip Arthroplasty Register;; e The Norwegian Arthroplasty Register, Department of Orthopaedic Surgery, Haukeland University Hospital, Bergen, Norway;; f Department of Clinical Medicine, University of Bergen, Norway;; g Coxa Hospital for Joint Replacement, and Faculty of Medicine and Life Sciences, University of Tampere, Tampere, Finland;; h Finnish Hip Arthroplasty Registry

## Abstract

Background and purpose — Mobilization has traditionally been restricted following total hip arthroplasty (THA) in an attempt to reduce the risk of dislocation and muscle detachment. However, recent studies have questioned the effect and rationale underlying such restrictions. We investigated the use of postoperative restrictions and possible differences in mobilization protocols following primary THA in Denmark (DK), Finland (FIN), Norway (NO), and Sweden (SWE).

Patients and methods — All hospitals performing primary THA in the participating countries were identified from the latest national THA registry report. A questionnaire containing questions regarding standard surgical procedure, use of restrictions, and postoperative mobilization protocol was distributed to all hospitals through national representatives for each arthroplasty registry.

Results — 83% to 94% (n = 167) of the 199 hospitals performing THA in DK, FIN, NO, and SWE returned correctly filled out questionnaires. A posterolateral approach was used by 77% of the hospitals. 92% of the hospitals had a standardized mobilization protocol. 50%, 41%, 19%, and 38% of the hospitals in DK, FIN, NO, and SWE, respectively, did not have any postoperative restrictions. If utilized, restrictions were applied for a median of 6 weeks. Two-thirds of all hospitals have changed their mobilization protocol within the last 5 years—all but 2 to a less restrictive protocol.

Interpretation — Use of postoperative restrictions following primary THA differs between the Nordic countries, with 19% to 50% allowing mobilization without any restrictions. There has been a strong tendency towards less restrictive mobilization over the last 5 years.

Dislocation following primary total hip arthroplasty (THA) has been reported to occur in 1–10% of patients (Meek et al. 2006, Patel et al. 2007, Kotwal et al. 2009, Jørgensen et al. 2014). Both surgery- and patient-related factors have been shown to affect the risk for dislocation, including surgical approach, implant position, implant type, implant fixation, femoral head size, age, sex, comorbidities, and cognitive function (Jolles et al. 2002, Byström et al. 2003, Brooks 2013, Seagrave et al. 2017a, 2017b, Miller et al. 2018, Tsikandylakis et al. 2018).

Movement restrictions and other hip precautions following THA have commonly been practiced to prevent dislocation and muscle detachment (Husted et al. 2014)—especially if a lateral transgluteal approach has been used. However, recent studies have questioned this rationale, as liberal postoperative mobilization protocols have been demonstrated not to increase the risk for dislocation (Peak et al. 2005, Restrepo et al. 2011, Gromov et al. 2015, Allen et al. 2018). This was confirmed by a recent systematic review, which concluded that a more liberal lifestyle restriction and precaution protocol did not increase the dislocation rates after THA (van der Weegen et al. 2015).

Despite increasing evidence that postoperative restrictions may be unnecessary, a recent study from the UK showed that 97% of physiotherapists and occupational therapists routinely prescribed hip precautions (Smith and Sackley 2016). Later, a national survey from the Netherlands found that restrictions were recommended to between 69% and 100% of patients following primary THA depending on the surgical approach used (Peters et al. 2017).

Little is known about the use of postoperative restrictions in the Nordic countries. Such knowledge of the utilization of postoperative restrictions would make it easier to compare and interpret studies from the different Nordic countries. This would also facilitate the investigation of complications and functional outcome following THA. Finally, with increasing evidence on limited benefits with postoperative restrictions, updated national guidelines are needed to reduce inequity in postoperative care following primary THA.

This questionnaire-based study investigated the use of postoperative restrictions and describes differences in mobilization protocols following primary THA in Denmark (DK), Finland (FIN), Norway (NO), and Sweden (SWE).

## Patients and methods

We identified all hospitals in DK, FIN, NO, and SWE performing primary THA from the respective national arthroplasty register’s most recent annual report.

A survey with questions regarding standard surgical procedure, use of restrictions, and postoperative mobilization was designed according to guidelines presented by Sprague et al. (2009). Besides asking if the hospitals used any mobilization restrictions at all following primary THA, we also asked about specific restrictions employed and specific aids given to the patients as a part of the standard mobilization protocol (Kornuijt et al. 2016, Lee et al. 2017). Authors KG, AT, and HH drafted the questionnaire, whereafter all other authors were asked to review it. Subsequently, we revised the questionnaire according to comments to increase clarity and face validity. The questionnaire was designed in English and is presented in Supplementary data. The questionnaire was distributed to all identified hospitals through national representatives for each participating arthroplasty registry by email or regular mail. The questionnaire was sent to the head of the arthroplasty department, who was asked to fill out the questionnaire on behalf of the department. If the head of the arthroplasty department was not identified, the questionnaire was sent to the head of the orthopedic department. Approximately 1 month after sending out the questionnaire, a letter or email was sent out to all non-respondents with a reminder to complete and return the questionnaire. Descriptive statistics were applied using IBM SPSS Statistics v25 (IBM Corp, Armonk, NY, USA).

### Ethics, funding, and potential conflict of interest.

No approval from the National Ethics Committee was necessary as this was a non-interventional observational study. No funding was received for this work. The authors declare no conflict of interest.

## Results

29, 33, 58, and 79 hospitals performing primary THA were identified in DK, FIN, NO, and SWE, respectively. 24/29, 27/33, 42/58, and 74/79 of the hospitals in DK, FIN, NO, and SWE, respectively, returned complete questionnaires giving a coverage of 84% (167/199). The hospitals responding to the questionnaire included 94% (45,440/48,386) of all primary THAs performed in Nordic countries in 2017 (Table 1).

**Table 1.** Coverage (n/N) of hospitals and procedures

	Coverage hospitals			Coverage procedures		
Country	n**^a^**	N**^b^**	(%)	n**^c^**	N**^d^**	(%)
Denmark	24	29		10,470	10,708	(98)
Finland	27	33		10,125	10,657	(95)
Norway	42	58		7,431	8,881	(83)
Sweden	74	79		17,414	18,140	(96)
Total	167	199	(84)	45,440	48,386	(94)

a number of hospitals performing primary THA that returned correctly filled out questionnaires.

b number of hospitals performing primary THA countrywide.

c number of primary THA performed annually in the hospitals that returned correctly filled out questionnaires.

d number of primary THA performed annually countrywide.

A posterolateral approach was the most common surgical approach in all countries. DK was the only country using elevated-rim acetabular components more frequently than neutral acetabular components (Table 2). 92% of the hospitals had a standardized mobilization protocol and 98% allowed immediate full weight-bearing (Table 2). 12/24, 11/27, 8/42, and 28/74 of the hospitals in DK, FIN, NO, and SWE, respectively, did not have any postoperative restrictions. For 31 hospitals that used different restrictions depending on the approach, 28 used restrictions for a posterolateral approach, 11 used restrictions for an anterolateral approach and 7 used restrictions for a direct lateral approach. 98% of the hospitals allowed immediate full weight-bearing.

**Table 2.** Demographics of hospitals that returned the correctly filled out questionnaire

	DK n = 24	FIN n = 27	NO n = 42	SWE n = 74	Total n = 167
Approach used					
Direct anterior	–	1	5	1	7
Anterolateral	1	9	8	36	54
Direct lateral	–	1	8	19	28
Posterolateral	23	27	31	48	129
Articulation					
Neutral	9	17	23	59	108
Highwall	15	10	19	15	59
Dual mobility	1	–	–	–	1
Standard protocol					
Yes	24	22	39	69	154
No	–	5	3	5	13
Full weight-bearing)					
Yes	23	27	40	74	164
No	1	–	2	–	3
Restrictions					
Yes	12	10	27	28	77
No	12	11	8	28	59
Depending	–	6	7	18	31
Aids					
Yes	10	18	20	52	100
No	14	9	22	22	67
Restriction changed within 5 years					
Yes	17	19	25	51	112
No	7	8	17	23	55
Supervised physiotherapy					
Yes	11	14	26	51	102
No	1	5	2	6	14
Individual	12	8	14	17	51

DK = Denmark; NO = Norway; FIN = Finland; SWE = Sweden.

**Figure 1.**
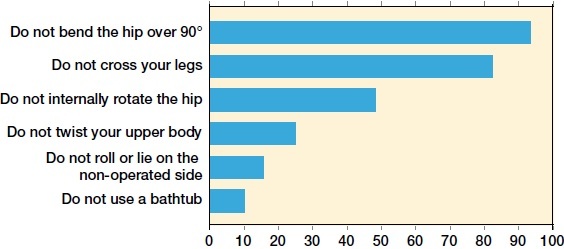
Restrictions used in hospitals that used restrictions.

**Figure 2.**
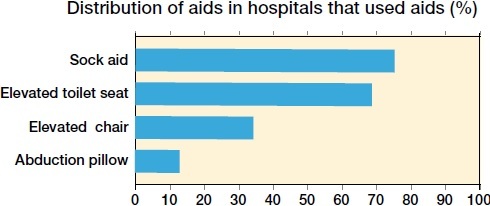
Aids used in hospitals that utilized aids on discharge.

For hospitals applying restrictions (n = 108), these were used for 2 weeks in 1%, for 4 weeks in 6%, for 6 weeks in 47%, and for a minimum of 12 weeks in 45% of the hospitals. Restrictions were used for a median of 6 weeks. As regards the approach used, 71% of the hospitals using a direct anterior approach did not use restrictions compared with 33% of hospitals using a posterolateral approach (Table 3). Avoiding bending the hip over 90 degrees and not crossing the legs were the most commonly employed restrictions (Figure 1). Aids (other than walking aids) were routinely used by 60% of the hospitals, with aids for putting on socks and an elevated toilet seat being the most common (Figure 2).

**Table 3.** Restrictions utilized depending on the approach

Restrictions	Direct anterior n = 7	Antero-lateral n = 54	Direct lateral n = 28	Postero lateral n = 129
Yes	2	28	14	86
No	5	26	14	43

67% (112/167) of all hospitals had changed their mobilization protocol within the last 5 years—all but 2 to a less restrictive protocol.

## Discussion

In this questionnaire-based study, we found that one-third of all participating hospitals did not use any postoperative restrictions following primary THA, while one-fifth imposed restrictions depending on the surgical approach. Denmark was the most liberal country with half of the hospitals not using any restrictions while Norway was the most restrictive country with one-fifth of the hospitals not employing any restrictions.

Very few previous studies have investigated the use of restrictions on a national level. Recently, Peters et al. (2017) performed a national survey investigating use of postoperative restrictions following primary THA in the Netherlands and found that restrictions were applied for between 69% and 100% of the patients depending on the surgical approach used. Based on our results, even the most conservative country in our study (NO) had a more liberal approach than the Netherlands. Our results also showed a more liberal approach compared with a survey from the UK, which found hip precautions to be routinely prescribed by 97% of health professionals (physiotherapists and occupational therapists) participating in the study (Smith and Sackley 2016). The median use of restrictions for 6 weeks reported in our study is in agreement with Peters et al. (2017), and Smith and Sackley (2016).

We found that restrictions were most frequently used with a posterolateral approach and least with a direct anterior approach. This conforms with the survey results by Peters et al. (2017) reporting a 100% restriction use with the posterolateral approach compared with 69% with the direct anterior approach. This difference is most likely explained by higher dislocation rates for a posterolateral approach compared with the direct anterior approach reported by some authors (Hailer et al. 2012, Zijlstra et al. 2017, Miller et al. 2018).

An important finding in our study is a strong trend towards a less restrictive mobilization protocol in recent years: two-thirds of hospitals had changed their mobilization protocol to a less restrictive one in the last 5 years. This is supported by the emerging evidence that removal of postoperative restrictions does not seem to lead to an increased risk for dislocation following primary THA (Peak et al. 2005, Restrepo et al. 2011, Gromov et al. 2015, van der Weegen et al. 2015, Kornuijt et al. 2016, Allen et al. 2018). Furthermore, a recent study showed that while most patients can remember all of the restrictions recommended at 8 weeks after surgery only one-fifth adhere to all restrictions, suggesting that even if restrictions are prescribed most patients do not adhere to them (Lee et al. 2017).

To our knowledge only 2 studies have found a correlation between removal of restrictions and increased risk of dislocation. In a registry-based study, Jørgensen et al. (2014) found that departments that did not use restrictions had a higher dislocation rate compared with departments that applied restrictions. However, the study was not designed to analyze restrictions, and the difference in dislocation rate could potentially be explained by a number of factors other than the use of restrictions. Mikkelsen et al. (2014) found a statistically nonsignificant increase in the dislocation rate in patients who were mobilized without restrictions, while no difference was seen in patient-reported outcomes after 6 weeks. However, this study investigated only 365 patients and was not powered to investigate dislocations following THA. Conversely, several authors have suggested that a liberal mobilization protocol following primary THA may lead to earlier return to work and higher patient satisfaction (Peak et al. 2005, Barnsley et al. 2015, van der Weegen et al. 2015).

Our study has several limitations. First, we asked about standard protocols in the participating hospitals; we do not know to what extent the individual surgeons adhered to these protocols. Second, while the response rate was excellent, 16% of the hospitals (accounting for 6% of annually performed THAs) did not respond, allowing for some degree of bias. There is, however, no obvious reason to think that the departments that did not reply systematically were less or more restrictive than the those that did so. Also, although we included a question regarding articulation size in our questionnaire, we did not investigate other factors that could influence the risk of dislocation such as the type of implant and fixation. These factors could affect the postoperative protocols (Kim et al. 2009, Seagrave et al. 2017a). In recent years there have been a trend towards increased use of larger femoral heads, as this has been suggested to reduce the risk for dislocation. A recent study from the Nordic Arthroplasty Registry Association found that the risk for revision due to dislocation was lower when comparing 32-mm heads to 28-mm heads, while no benefit with use of 36-mm heads over 32-mm heads was found (Tsikandylakis et al. 2018). We did not investigate the femoral head size used by the individual departments, and this could potentially affect the surgeons in regard to use of postoperative restrictions. Further, we did not investigate whether or not the use of restrictions differed depending on the diagnosis. While we have investigated the restrictions recommended by the hospitals, we do not know if the physiotherapists working with the patients after discharge adhered to those protocols. Finally, while we investigated the use of restrictions, we did not investigate the dislocation rates, patient-reported outcomes, or patient satisfaction in departments using restrictions or in those with a more liberal mobilization protocol. Thus, no conclusion can be drawn from this study on the potential association between the use of postoperative restrictions and dislocation rates following primary THA.

In summary, we found that use of postoperative restrictions following primary THA differed between the Nordic countries with 19–50% allowing mobilization without any restrictions. There has been a strong tendency towards less restrictive mobilization over the last 5 years. Whether this trend has had any affect on the dislocation rates and whether the dislocation rates differ between the less and the more restrictive hospitals in Nordic countries is unknown.

### Supplementary data

The questionnaire is available as supplementary data in the online version of this article, http://dx.doi.org/ 10.1080/17453674.2019.1572291

## Supplementary Material

Supplemental Material
