# Supplementary material for: Varying but reduced use of postoperative mobilization restrictions after primary total hip arthroplasty in Nordic countries: a questionnaire-based study
Source: Acta Orthop. 2019 Feb 11;90(2):143–7. doi: 10.1080/17453674.2019.1572291 (PMC6461082; doi:10.1080/17453674.2019.1572291)
Supplement: Supplemental Material [file IORT_A_1572291_SM4247.pdf]

Supplementary data

Questionnaire regarding use of postoperative mobilisation restrictions following Primary Total Hip Arthroplasty

Dear colleague

We are investigating the use of postoperative restrictions following Primary Total Hip Arthroplasty. This is a collaboration project between Denmark, Sweden, Norway and Finland investigating the nationwide use of postoperative restrictions in Nordic countries following Primary Total Hip Arthroplasty. We hope you could spend 5 minutes answering this questionnaire.

We thank you in advance for participating.

Any questions can be addressed to XXXXXXX

Please return this questionnaire by regular mail or email to:
XXXXXX

1) What approach does you department use (several answers possible)?
   - ☐ Direct anterior
   - ☐ Anterolateral
   - ☐ Direct lateral
   - ☐ Posterolateral

2) What is your standard articulation choice for the acetabular component in a primary THA (the one you use for the majority of your primary THAs)?
   - ☐ Neutral liner / cup
   - ☐ Highwall /elevated / 10 degree liner / cup
   - ☐ Dual mobility

3) Do you have a standard mobilisation protocol that is followed by all hip surgeons?
   - ☐ Yes
   - ☐ No – postoperative mobilization protocol based on individual surgeons preference.

4) Do you allow full weight bearing on the day of surgery?
   - ☐ Yes
   - ☐ No

   If "no" – when do you allow full weight bearing?
   - ☐ after minimum 2 weeks
   - ☐ after minimum 4 weeks
   - ☐ after minimum 6 weeks
   - ☐ after minimum 8 weeks

5) Do you have any postoperative mobilization restrictions?
   - ☐ Yes
   - ☐ No
   - ☐ Depending on the approach

   If "depending on the approach" please note for which approach are any restrictions used?
   - ☐ Direct anterior
   - ☐ Anterolateral
   - ☐ Direct lateral
   - ☐ Posterolateral

6) What hip restrictions do you inform you patients about: (several options possible)
   - ☐ Do not bend your hip more than 90 degrees
   - ☐ Do not cross your legs or feet
   - ☐ Do not roll or lie on your unoperated side
   - ☐ Do not twist your upper body when standing
   - ☐ Do not internally rotate the hip when turning
   - ☐ Sleep on your back
   - ☐ Do not use a bath (tub)
   - ☐ Use aids to put on underwear/socks/shoes

   For how long are restrictions applied? If different times for each restrictions, list the longest
   - ☐ minimum 2 weeks
   - ☐ minimum 4 weeks
   - ☐ minimum 6 weeks
   - ☐ minimum 12 weeks

 Acta Orthopaedica 2019; 90 (DOI: 10.1080/17453674.2019.1572291)

7) What aids are given to all patients as standard?
   - ☐ None
   - ☐ Abduction pillow while sleeping
   - ☐ Elevated toilet seat
   - ☐ Elevated chair
   - ☐ Aid for putting on shoes/socks

8) Do you recommend using crutches or other walking aids to all patients (as standard)?
   - ☐ Yes
   - ☐ No – only if needed

   If "yes" – for how long do you recommend that patients use walking aids?
   - ☐ only as long as the patient feels the need for it
   - ☐ minimum 2 weeks
   - ☐ minimum 4 weeks
   - ☐ minimum 6 weeks
   - ☐ minimum 12 weeks

9) Who informs the patients regarding the restrictions lack of restrictions (several options possible)?
   - ☐ Surgeon / Ward doctors
   - ☐ Nurse
   - ☐ Physiotherapist

10) Do patients receive supervised physiotherapy following discharge?
   - ☐ No
   - ☐ Some – based in individual assessment
   - ☐ All

   If "some" or "all" for how long do they receive supervised therapy?
   - ☐ minimum 2 weeks
   - ☐ minimum 4 weeks
   - ☐ minimum 6 weeks
   - ☐ minimum 12 weeks
   - ☐ according to the physiotherapist's assessment

11) What is your median length of stay for primary THA at your department?
   - ☐ 1–2 days
   - ☐ 2–4 days
   - ☐ 5–7 days
   - ☐ >7 days

12) Have you changed your mobilization protocol within the last 5 years?
   - ☐ Yes
   - ☐ No

   If "yes" did the changes make the postoperative mobilization protocol?
   - ☐ More restrictive
   - ☐ Less restrictive

Thank you for taking time to answer the questions.

Please return this questionnaire by regular mail or email to:
XXXX
